# Historical Development and Experience of Day Surgery in China: From the Perspective of Anesthesiologists

**DOI:** 10.1111/pan.15078

**Published:** 2025-02-07

**Authors:** Jiangrong Luo, Chunbao Xie, Dan Fan

**Affiliations:** ^1^ Department of Anesthesiology, Ambulatory Surgery Center, Sichuan Provincial People's Hospital University of Electronic Science and Technology of China Chengdu China; ^2^ Department of Laboratory Medicine and Sichuan Provincial key Laboratory for Uman Disease Gene Study, Sichuan Provincial People's Hospital University of Electronic Science and Technology of China Chengdu China

**Keywords:** ambulatory surgery center, anesthesiologists, China, day surgery, historical development, management model

## Abstract

**Background:**

Day surgery has become the main mode of surgery in American and European countries, but it is still in the early stage in developing countries due to the limitation of medical technology and the backward management concept. At present, day surgery accounts for more than 60% of elective surgery in many countries in Europe and North America and more than 85% in countries such as the United Kingdom and the United States. There are 8469 ambulatory surgery centers in the United States in 2023. In China, the first ambulatory surgery center was established in 2001. In 2018, more than half of the tertiary hospitals (high‐level hospitals) in China carried out day surgery, of which 639 hospitals set up ambulatory surgery centers; the proportion of day surgery in elective surgery increased to 12.8%. The annual number of day surgeries exceeded 1.25 million. In 2022, our hospital established an ambulatory surgery center managed by anesthesiologists. Day surgery requires anesthesiologists to participate in the whole process of patient management from preoperative preparation to postoperative recovery. The establishment of ambulatory surgery centers managed by anesthesiologists is of great significance to China, developing countries, and the whole world.

**Objectives:**

So this study aimed to review the development of day surgery in China, combine Chinese government policy evolution, summarize the management model of Chinese ambulatory surgery centers, guide the establishment of ambulatory surgery centers in low‐ and middle‐income countries, and highlight and analyze the advantages of anesthesiologist‐managed ambulatory surgery centers as distinct from other physician‐managed ones.

**Discussion:**

We call on anesthesiologists, other physicians, surgeons, nurses, and health system managers around the world to promote efficient, low‐cost day surgery in developing countries and thereby increase access to surgical treatment for the world's poor.

**Abbreviations**
AS: ambulatory surgery
ASA: American Society of Anesthesiologists
ASC: ambulatory surgery center
ASCs: ambulatory surgery centers
DS: day surgery
ERAS: enhanced recovery after surgery
PADS: postanesthesia discharge score
PDNV: postdischarge nausea and vomiting
PONV: postoperative nausea and vomiting

## 1 Introduction

Day surgery (DS) or ambulatory surgery (AS) is generally defined as the surgery performed within 1 day of a hospital stay. In 2003, The International Association for Ambulatory Surgery (IAAS) defined DS as a procedure in which the patient is admitted, operated, and discharged within one working day, with the exception of outpatient surgery performed in a physician's office or hospital. The definition of DS in Great Britain and Ireland is that the patient is admitted and discharged on the same day, with DS as the intended management. The term “23‐h stay” should be avoided; this is used in the US health care system [1]. The Chinese Ambulatory Surgery Alliance defines DS as a planned surgery that is performed, and the patient is discharged within 24 h [2]. Table 1 lists some data about AS centers (ASCs) between the United States and China [2, 3, 4].

**TABLE 1:** Comparison of data about ambulatory surgery centers between the United States and China.

Data about ambulatory surgery centers	The United States	China
Time when ambulatory surgery center was first established	1970s	2001
Number of ambulatory surgery centers	7447 in 2019 (no exact data were reported in 2018) 8469 in 2023	639 in 2018 The number is increasing rapidly, but no exact data were reported in 2023
Annual number of day surgeries in ambulatory surgery centers	23 million in 2023	1.25 million in 2018
Proportion of day surgery in elective surgery	More than 85% in 2023	12.8% in 2018
Directors and resident physicians of ambulatory surgery centers	Surgeons, general physicians, or anesthesiologists	Surgeons, general physicians, or anesthesiologists
Ownership of day surgery centers	Most are independent of hospitals	Attached to large public hospitals
Processes	Standardizing practices to enhance outcomes and manage costs	Standardizing practices to enhance outcomes and manage costs
Fees	Procedures performed in ambulatory surgery centers cost 45% (on average) less than those performed in hospitals	Procedures performed in ambulatory surgery centers cost 35% (the maximum reduction) less than those performed in our hospital's other departments. (Data were obtained from our hospital and no national data were reported.)

Independent ASCs emerged in the United States in the 1970s. Evidence indicates that the costs in the ambulatory setting can be significantly lower than those in the hospital setting. ASCs have superior efficiency and productivity [5]. Therefore, DS has become the main mode of surgery in American and European countries, but it is still in the early stage in developing countries due to the limitation of medical technology and the backward management concept [6]. However, the deficiencies in the development of DS in China are not the focus of this paper.

Economic development in China and other developing countries in the 21st century has been accompanied by the continuous expansion of people's demand for quality medical services. Evidence suggests that performing DSs has several merits benefiting the patient, the hospital, and the medical system [7, 8, 9]. Hence, the number of DSs has increased rapidly in the last two decades in China.

The medical environment, policy environment, and cultural traditions of developing countries are quite different from those of developed countries. This study aimed to review the development of ASCs in China, combine the excellent experience of developed and developing countries, and guide the establishment of ASCs in low‐ and middle‐income countries. This study also highlighted and analyzed the advantages of anesthesiologist‐managed ASCs as distinct from other physician‐managed ones.

## 2 Policy Evolution of DS and Establishment Process of ASC in China

As the advantages of DS were gradually recognized in developing countries, DS was introduced spontaneously by a few medical institutions in China at the end of the 20th century. However, traditional culture (both doctors and patients are accustomed to surgical treatment needs to be carried out in the inpatient unit until the patient has fully recovered), economic underdevelopment, and concerns about surgical safety affected the general public's acceptance of DS. Government policy support was essential for medical reform in low‐ and middle‐income countries. The acceptance of DS was low without policy support. The evolution of the policy was divided into three stages: policy gap stage, policy exploration stage, and policy support stage [10]. These stages revealed the following rules: First, the introduction of new things is often carried out spontaneously by the people, and medical institutions in developing countries cannot wait passively for the policy support of the government. The achievements of hospitals in DS can increase confidence and encourage the government to take the initiative to support DS. Second, the governments of developing countries should introduce policies on medical insurance, infrastructure support, medical standards, and performance appraisal as soon as possible to promote the rapid and healthy development of DS. Health insurance policies should be introduced first for low‐ and middle‐income countries. China has a national unified government medical insurance with low premiums and basic medical benefits covering the entire population. Only a small number of people with better economic conditions will buy commercial insurance on their own, which is not provided by the government.

**FIGURE 1:**
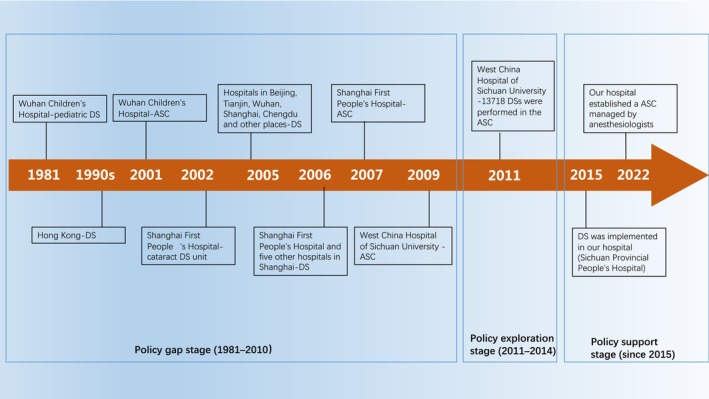
Important time nodes in the development of DS in China. ASC, ambulatory surgery center; DS, day surgery.

Figure 1 shows the important time nodes in the development of DS and the policy changes in China to help developing countries understand how to carry out the building of ASC in low‐ and middle‐income countries.

### 2.1 Policy Gap Stage (1981–2010)

- Wuhan Children's Hospital began 1‐day pediatric surgery in 1981, thereby setting up a precedent for Chinese doctors.
- In Hong Kong, DS has been carried out since the 1990s [11]. Hong Kong had mirrored the approaches in Great Britain and Australasia prior to reunification, and Hong Kong returned to China in 1997.
- In 1997, some academic discussions were made on DS in Chinese academic journals [12], but no attention was paid at that time.
- In 2001, ASC was formally established to centralize treatment and management in Wuhan Children's Hospital. It was the earliest documented ASC in China [13].
- In 2002, Shanghai First People's Hospital started ambulatory cataract surgery and set up an independent cataract DS unit in the following year [14].
- Since 2005, the hospitals in Beijing, Tianjin, Wuhan, Shanghai, Chengdu, and other places have carried out DS in succession; although the number of surgeries was still small, the momentum was clear [10, 15].
- In particular, it should be noted that Shanghai First People's Hospital and five other hospitals in Shanghai gradually performed DS in 2006 and achieved significant success under the unified coordination of the Shanghai Shenkang Hospital Development Center. There was governmental financial support in the establishment of the center [16].
- In 2007, a more functional ASC was established in the Songjiang branch of Shanghai First People's Hospital. This was the first ASC in China as reported in previous studies, including consultation service areas, wards, and independent operating rooms [10]. The development of DS in Shanghai was at the forefront of the country during this period. The reason was that despite no direct support of government policy during the promotion of DS in Shanghai, the Shanghai Shenkang Hospital Development Center represented the support and promotion of the government to some extent.
- The ASC of West China Hospital of Sichuan University was opened in 2009 [15].

DS was first carried out by doctors spontaneously in China, similar to the developed countries, and was first attempted by doctors in Wuhan Children's Hospital in 1981, without policy support. At this stage, the government insurance only paid for the expenses incurred by the inpatient unit, and the government did not issue a document declaring its support for DS in all respects. What was the driving force for the beginning of DS in China in the last about 30 years from 1981 to 2010? Primarily, China has a large population, an underdeveloped economy, and inadequate primary care. China and the United States formally established diplomatic ties in 1978, followed by China's reform and opening up. Since then, the people's demand for health care services has increased accordingly with the improvement in their living standards. As a result, primary medical institutions could not meet the needs of patients, and patients were concentrated in large hospitals in big cities. This directly led to long waiting times for hospitalization and surgery. Large hospitals could not cope with the pressure by expanding their beds. The doctors began to pay attention to DS to improve the efficiency of hospital operations.

### 2.2 Policy Exploration Stage (2011–2014)

x By July 2011, 13 718 DSs were performed in the ASC of West China Hospital of Sichuan University. The departments participating in the surgeries were ophthalmology, ear, nose, and throat surgery, general surgery, thyroid surgery, breast surgery, pediatric surgery, gastroenterology, hepatobiliary surgery, vascular surgery, urology, nephrology, orthopedics, and so forth. The surgical procedures included laparoscopic cholecystectomy, hernia repair, cataract extraction, polypectomy of the vocal cord, varicose ligation of the great saphenous vein, most pediatric surgeries, lumpectomy of the breast, choledochoscopic surgery, laparoscopic peritoneal dialysis catheter placement, and minimally invasive surgery in urology [15].
xi Since 2011, the emergence of government policies supporting the development of DS has predated the emergence of local DS in many parts of China. These government policies guided the promotion of DS. For example, a large number of hospitals in Jiangsu Province were encouraged to start DS in 2013. At the same time, a large number of hospitals in areas without government policy support were encouraged to start DS. These hospitals were from Shanxi, Guangdong, Shandong, Zhejiang, Jilin, Hunan, Xinjiang, and other provinces [10].

From 2011 to 2014, as the medical institutions in China performed more DSs, detailed medical data and powerful evidence were presented to the government. Some medical institutions used these data and evidence to persuade local governments to issue policies supporting DS. Although the central government did not issue policies to support DS during this period, many local governments began to actively support the development of DS. Some local government health insurance covers the cost of DS.

For instance, the ASC of West China Hospital of Sichuan University was opened in 2009 in Chengdu City. By July 2011, 13 718 DSs were performed in the ASC of West China Hospital of Sichuan University [15]. Since then, the evidence of its successful operation has convinced the medical insurance administration to reach a consensus that DS can greatly improve the utilization rate of medical insurance funds and avoid waste. In 2011, the Chengdu Municipal Government issued a document requiring more than 85% of public hospitals in Chengdu to perform DS to promote the equalization of public health services [10].

At the same time, national academic organizations were established. With the joint efforts of Shanghai Shenkang Hospital Development Center, West China Hospital, Xiangya Hospital, and other domestic institutions, the China Ambulatory Surgery Alliance was officially established in Beijing in 2012. In 2013, the alliance officially represented China as a member of the IAAS. In 2013 and 2014, the first and second national academic annual conferences of DS were organized in China.

### 2.3 Policy Support Stage (Since 2015)

xii In 2015, DS was implemented in our hospital (Sichuan Provincial People's Hospital, University of Electronic Science and Technology of China) [17].
xiii In 2022, our hospital established an ASC managed by anesthesiologists.

Since 2015, the national policies on medical insurance, infrastructure support, medical standards, and performance appraisal have greatly promoted the development of DS, and the inclusion of DS in medical insurance nationally has also removed the biggest obstacle to its expansion. Therefore, with the promotion of the national government policy in 2015, our hospital also joined the ranks of DS. Table 2 lists the vital national government policies to promote the popularization of DS in China [18].

**TABLE 2:** Vital national government policies to promote the popularization of day surgery in China.

Release time	Policy name	Main content	Key point
2015	National Health Commission of the People's Republic of China and the National Administration of Traditional Chinese Medicine Concerning the Notice of the Action Plan to Further Improve the Medical Service ([2015] No. 2)	Day surgery was proposed as a measure for the rational allocation of medical resources	Infrastructure support
2015	Guidelines of the General Office of the State Council on the Comprehensive Reform of Urban Public Hospitals ([2015] No. 38)	It was proposed to gradually expand the medical insurance payment for day surgery	Medical insurance
2016	National Health Commission of the People's Republic of China and the Ministry of Human Resources and Social Security of the People's Republic of China on the Pilot Work Plan for Day Surgery in Tertiary Hospitals ([2016] No. 306)	The pilot work of day surgery in tertiary hospitals was launched, and a number of diseases and surgical methods for day surgery were recommended in the annexure	Medical standards
2017	Notice of the National Health Commission of the People's Republic of China on Issuing Key Points of Health and Family Planning Work in 2017 ([2017] No. 11)	“Expanding the scope of day surgery” was included as a key task in 2017	Infrastructure support
2019	General Office of the State Council Concerning the Deepening Medical and Health System Reform in 2019 Key Tasks ([2019] No. 28)	Tertiary public hospitals were encouraged to actively adjust the outpatient disease structure and gradually expand the diseases of day surgery	Performance appraisal
2020	Notice of the General Office of the National Health Commission on the Issuance of the First Batch of Specifications for Day Surgery ([2020] No. 1)	The criteria of admission and discharge, main operation, and medical service items of day surgery were stipulated	Medical standards

Based on the development of ASCs in China, the rules can be summarized as follows.

First, developing countries should start with simple DSs, and gradually increase the level of surgery and perform more types of surgeries.

Second, In China, most patients prefer to have surgery done in the inpatient department and hope that the hospital stay can be extended until the healing time of the surgical incision. This is due to patients' concerns about underdeveloped medical technology and the lack of adequate community health care system when they return home. Even a few patients live days away from the nearest hospital. Hence, a simple DS should be pioneered in each department of a large hospital in big cities in developing countries. For example, a room was first created in the ophthalmic inpatient unit for cataract DS. This should ease patients' concerns about underdeveloped medical technology and ease medical staff's concerns about the uncertainty of the risks of new DS. After the safety of DS is guaranteed and the process is mature, DS wards for certain diseases can be established, and finally, an independent ASC can be established. It is worth noting that ASCs in China are currently attached to large public hospitals. This is because medical staff in large public hospitals first tried to introduce DS into China, and then the government first gave policy support to DS in large public hospitals. Government health insurance is also more willing to pay for DS at large public hospitals.

Third, the management model of perioperative period has gone through three stages: [14]

- Decentralized admission and decentralized treatment:

It means that the whole process of admission and treatment is dispersed in each inpatient ward, and there is no unified management department and full‐time management staff in the hospital. In this stage, each clinical department arranges some beds as DS beds, runs the management according to the clinical pathway of DS, shares the operating room of the inpatient ward with ordinary surgical patients, and makes the appointment of surgery schedule and postoperative follow‐up by each department. There are no full‐time staff (from front office staff to the nurses, physician assistants, residents, attending surgeons, and anesthesiologists) to manage DS. They need to manage both DS patients and other surgery patients in the same ward.

The advantage of this is that in a hospital that is just experimenting with DS, there is no need to deploy specialized medical staff to manage DS.

ii Centralized admission and decentralized treatment:

“Centralized admission” means that the appointment schedule and postoperative follow‐up of DS are uniformly carried out by the full‐time staff. The hospital has a unified management of DS appointment and follow‐up centers.

“Decentralized treatment” means that there are also no full‐time staff (nurses, physician assistants, residents, attending surgeons, and anesthesiologists) to manage DS. They need to manage both DS patients and other surgery patients in the same ward and share the operating room of the inpatient ward with ordinary surgery.

This stage occurs because centralized admission is easier to complete than centralized treatment, so it is a transitional stage.

iii Centralized admission and centralized treatment:

It refers to the establishment of an independent ASC as a centralized management platform. The appointment schedule and postoperative follow‐up of DS are uniformly carried out by the full‐time staff. There are also full‐time staff (nurses, physician assistants, residents, attending surgeons, and anesthesiologists) to manage DS. DS patients have a separate ward and operating room from other surgery patients.

## 3 ASC Managed by Anesthesiologists

**FIGURE 2:**
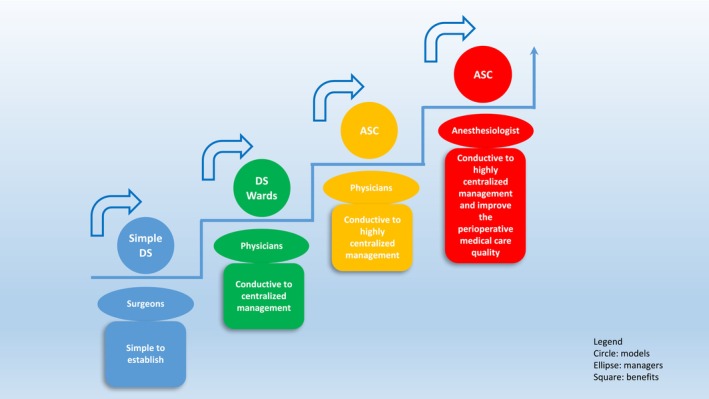
Different management models and their managers. DS, day surgery; ASC, ambulatory surgery center.

Different management models require different managers (Figure 2). Usually, a simple DS was pioneered in each department of surgery in China. For example, cataract DS was initially performed in an ophthalmology ward, not in an ASC. Therefore, the managers of DS were first and foremost surgeons. After the safety of DS was guaranteed and the process was mature, DS wards for certain diseases were established, and finally, an independent ASC was established. At this point, the physicians who were internists, usually acting as the directors and staff physicians of the DS ward or ASC, managed patients in the perioperative period and did not participate in surgery, began to manage the wards, allowing the surgeons to concentrate only on performing the huge volume of surgeries in the operating room. However, higher requirements were put forward for perioperative anesthesia management with the development of surgical techniques and the shortening of hospital discharge time. ASCs managed by anesthesiologists who usually act as the directors and staff physicians of the DS ward or ASCs gradually emerged in China. There are no official data on how many DS centers are managed by anesthesiologists in China now.

As one of the largest hospitals in China, our hospital performs about 2000 DSs every month. In 2022, our hospital established an ASC managed by anesthesiologists. In the following text, the ASC in our hospital is used as an example to illustrate how to set up an ASC managed by anesthesiologists. The infrastructure of our ASC includes an appointment reception center, a ward, operating rooms, and anesthesia recovery rooms. When these facilities are established, they need the appropriate medical staff to operate them.

- In our appointment center, the staff is required to make the surgery appointment and the anesthesiologist to perform the preoperative anesthesia assessment. The surgeon only needs to leave these appointments and assessments to the staff and anesthesiologist.
- In our ward, operating rooms, and anesthesia recovery rooms, Anesthesiologists not only ensure the patient's perioperative painless and anesthesia‐related safety but also play a crucial role in perioperative medical care, especially in promoting the patient's postoperative recovery. Surgeons can more easily focus solely on the surgical procedure.

The specific processes involved in anesthesiologist‐led DS and the unique advantages of anesthesiologists in managing ASC are as follows.

### 3.1 Anesthesiologists Scheduling

In China and many developing countries, there is a scarcity of anesthesiologists. By the end of 2022, according to the official website of the National Health Commission, there were only 76 000 anesthesiologists in China, with only one anesthesiologist for every 180 000 people. In China, it is difficult to carry out anesthesiologists scheduling. As the person in charge of the ASC, the anesthesiologist can schedule the anesthesiologist more conveniently.

### 3.2 Preoperative Assessment

In the anesthesia clinic, preoperative assessment mainly includes history collection, American Society of Anesthesiologists (ASA) grading, Mallampati airway grading, cardiopulmonary function grading, etc. ASA III or above patients with severe complications and poor control of coexisting diseases are usually not suitable for DS.

For patients with complex physical conditions, anesthesiologists of our ASC will initiate a multidisciplinary consultation to optimize preoperative management, such as cardiology, respiratory, and nutrition. Before (managers were surgeons or general physicians) the establishment of our ASC, anesthesiologists would not initiate a multidisciplinary consultation, but the patients themselves went to the outpatient department for consultation. Now, patients can receive multidisciplinary consultation only in the anesthesia clinic, which can reduce the cancellation rate of surgery, avoid the waste of resources [19], and reduce the medical cost and mortality of patients [20].

After the establishment of our ASC, the cancellation rate on the day of surgery decreased from 3.9% to 2.5%.

### 3.3 Preoperative Education

The main contents of preoperative education (done by the surgeon after admission before the establishment of our ASC and done by the anesthesiologist in the anesthesia clinic after the establishment of our ASC):

- The proposed anesthesia and surgery plan;
- Preoperative and postoperative precautions, such as smoking and drinking cessation before surgery, appropriate exercise, early postoperative eating, and early ambulation;
- The management process of DS.

After anesthesiologists become managers of our ASC, they become more active in understanding the operation management process outside the operating room and more comprehensively communicate with patients. Anesthesiologists educate patients in the anesthesia clinic through oral, brochures, exhibition boards, multimedia, and Internet which will save the cost of transportation and accommodation for patients and improve patient satisfaction [21, 22]. Good preoperative education can make patients fully understand the enhanced recovery after surgery (ERAS) process, clarify their role in the process of diagnosis and treatment, improve the sense of participation, effectively relieve anxiety, improve compliance, and shorten the length of hospital stay [23, 24].

In our ASC, 100% of the patients received preoperative education by the anesthesiologist in the anesthesia clinic, which reduced the rate of patient reconsultation on these contents from 66.7% (before ASC establishment) to 16.7% (after ASC establishment) after admission.

### 3.4 Premedication and Preventive Analgesia

- Premedication: Short‐acting benzodiazepines are used for sedation in patients with a high anxiety state or difficulty cooperating with anesthesia procedures by anesthesiologists of our ASC.
- Preventive analgesia: Nonopioid analgesics can be given for preventive analgesia by anesthesiologists of our ASC.

Before the establishment of our ASC, there was no plan for premedication and preventive analgesia for DS. Preoperative chronic pain can lead to anxiety, depression, and other psychological problems, and affect the intensity of postoperative pain [25, 26]. Anesthesiologists of our ASC can find and observe more patients' preoperative anxiety and pain than just staying in the operating room. They have adequate knowledge of sedation and analgesia and do not have to worry about affecting the intraoperative anesthesia process. Recent evidence shows that the appropriate use of midazolam before surgery does not increase the risk of postoperative delirium [27, 28]. However, surgeons or general physicians may have concerns about the use of sedative and analgesic drugs before surgery.

The percentage of patients at our ASC who reported preoperative anxiety and pain declined from 5.3% and 5.1% in the first month of establishment to 2.1% and 1.9% in 2024, respectively.

### 3.5 The Choice of Anesthesia Method and Anesthetic Drugs

The concept of ERAS requires that the anesthesia protocol with the fastest recovery and highest comfort should be selected on the basis of ensuring the safety of patients and meeting the needs of surgery. Compared with local anesthesia, general anesthesia has the characteristics of shorter operation time and intraoperative ignorance, which has been widely used in DS anesthesia. Laryngeal mask airway has the characteristics of less airway irritation and less pharyngeal discomfort than tracheal intubation. Therefore, general anesthesia under laryngeal mask airway can be the first choice for DS patients without the risk of reflux and aspiration or special position requirements [29].

Patients with DS have a short hospital stay, so it is necessary to choose drugs with rapid onset, high clearance rate, and less effect on liver and kidney function. Etomidate, propofol, remifentanil, sevoflurane, and desflurane are suitable for anesthesia in DS [30]. For surgery requiring muscle relaxation or patients requiring tracheal intubation, short‐acting and medium‐acting nondepolarizing muscle relaxants such as cisatracurium and rocuronium can be used.

General anesthesia and local anesthesia accounted for 95.5% and 4.5% of all DS in our ASC, respectively. So far, except for PONV and postoperative pain, no other adverse reactions directly related to anesthesia have been recorded.

### 3.6 Lung‐Protective Ventilation Strategies

The protective pulmonary ventilation strategy in ERAS process is the key measure to reduce postoperative pulmonary complications, especially suitable for patients with high risk of postoperative pulmonary complications such as age > 50 years, body mass index > 40 kg/m^2^, ASA physical status>II, OSA, anemia, hypoxemia, and positive pressure ventilation time > 2 h [31].

The surgeon is not able to supervise the anesthesiologist during the procedure and to demonstrate a direct relationship between postoperative complications and the failure to perform lung‐protective ventilation strategies. Anesthesiologists, as the main body of managing DS patients, can be more active and strict in implementing lung‐protective ventilation strategies.

### 3.7 Perioperative Fluid Management

Before the establishment of our ASC, anesthesiologists could only manage the intraoperative fluid. After the establishment of our ASC, anesthesiologists can manage preoperative, intraoperative, and postoperative fluids. Appropriate perioperative fluid management can reduce the incidence of postoperative complications, and promote the recovery of lung function and motor function.

Goal‐directed fluid therapy is a way of fluid management based on the hemodynamic parameters of patients. Studies have shown that it can reduce postoperative complications in critically ill patients, so it is widely used in clinical practice [32]. However, regardless of the situation, tissue perfusion should be maintained first, and the minimum requirement is to maintain the mean arterial pressure above 65 mmHg or within 10%–20% of the preoperative baseline value [33].

### 3.8 Temperature Management

The harms of accidental hypothermia during perioperative period include incision infection, changes in coagulation function, shivering, myocardial ischemia, arrhythmia, delayed recovery from anesthesia caused by prolonged or altered drug effects, and increased mortality [34, 35, 36, 37]. The risk factors for accidental hypothermia during perioperative period are anesthesia and operation time > 2 h, age > 60 years, overweight, high ASA grade, major surgery, endoscopic surgery, intraoperative blood loss > 300 mL, and unheated infusion > 1500 mL [35, 38].

Chinese ERAS guidelines emphasize that all patients should routinely check their body temperature during surgery until after surgery to guide intraoperative temperature management. It is necessary for anesthesiologists to take the lead in promoting intraoperative temperature management. However, many hospitals in China lack active heating methods and equipment. A survey of several hospitals in Beijing in 2015 showed that only 11% of patients received intraoperative active warming [39], while a national sampling survey in 2017 found that the incidence of perioperative hypothermia (core body temperature < 36°C) reached 44.3% [40].

After the establishment of our ASC, we implement temperature protection in all our patients. The anesthesiologist prewarmed the patient before operation, took the method of passive forced‐air warming, and continued air warming during the operation.

### 3.9 Management of Pain

Before the establishment of our ASC, surgeons used only one analgesic drug after surgery. Intraoperative analgesia is led by anesthesiologists. Now, it is the policy of our ASC that anesthesiologists must use multimodal analgesia, which is a combination of different pharmacological and nonpharmacological interventions that can provide superior pain relief than a single agent.

Before the establishment of our ASC, in order to save time and manpower, anesthesiologists may not use local infiltration and peripheral nerve block techniques.

Now, It is the policy of our ASC that anesthesiologists must use these techniques in appropriate procedures.

The percentage of patients at our ASC who reported postoperative pain declined from 20.7% in the first month of establishment to 4.1% in 2024.

### 3.10 Management of Postoperative Nausea and Vomiting (PONV) and Postdischarge Nausea and Vomiting (PDNV)

PONV is one of the most common anesthesia‐related complications in the perioperative period. Severe nausea and vomiting are closely related to wound dehiscence, electrolyte disturbance, and other complications, which can lead to prolonged hospitalization and increased medical costs, and are also the main reason for decreased patient satisfaction.

Compared with elective major surgery, DS is less traumatic, and with the application of ERAS concept, the use of opioids and the time of fasting and water deprivation are significantly reduced, so the incidence of PONV may be lower. However, patients undergoing DS are at risk of PDNV. Apfel et al. [41] pointed out that the incidence of PONV in DS patients in the postanesthesia care unit was 20.7%, while the incidence of PDNV was 37.1%. These patients with PDNV are usually difficult to obtain rapid and effective intravenous antiemetic drug intervention immediately, which may lead to serious consequences such as reflux and aspiration.

In our ASC, PONV and PDNV scores are used to assess the risk of PONV and PDNV before surgery by anesthesiologists. The latest PONV guidelines recommend multimodal PONV prevention for adult patients with one or more risk factors. For minor patients, prevention is recommended for those with medium and high risk.

Our measures to prevent PONV are as follows:

xiv Ondansetron orally dissolved membrane was used before and after surgery;
xv Chewing gum was performed before and after surgery;
xvi The anesthesiologist administered dexamethasone 5 mg intravenously before induction of anesthesia and tropisetron 5 mg intravenously before the end of surgery;
xvii Adequate analgesia, early feeding, and early ambulation were required;
xviii For patients with PONV prophylaxis failure, antiemetic therapy with drugs with different mechanisms than prophylactic drugs is used (like metoclopramide), and such patients are encouraged to continue to receive prophylactic antiemetic therapy after discharge. Our patient received 8 mg of ondansetron orally dissolved membrane on postoperative Day 1 and Day 2.

Through these measures, the incidence of PONV and PDNV at our ASC declined from 20.1% and 0.2% in the first month of establishment to 5.5% and 0.1% in 2024, respectively. The reason for the low incidence of PDNV is that we treated patients with PONV aggressively. At the time of discharge, nausea and vomiting were almost nonexistent. In China, if significant symptoms are present at discharge, patients will refuse to be discharged, given the scarcity of medical resources outside the hospital.

### 3.11 Postoperative Nutritional Support and Early Mobilization

Early postoperative eating and drinking can promote the recovery of intestinal function and shorten the length of hospital stay. Nutan et al. [42] found that early postoperative water intake can reduce the incidence of PONV and opioid consumption in pediatric patients without increasing any adverse events.

Prolonged bed rest after surgery will increase adverse reactions such as thromboembolism, pneumonia, atelectasis, and muscle weakness. As one of the perioperative nursing measures strongly recommended by ERAS concept, early postoperative mobilization can effectively prevent the above adverse reactions [43].

However, due to the obstacles of urinary catheter, infusion line, and postoperative pain factors, patients' compliance with this measure is poor at present. In our ASC, tubule‐free technique, multimodal and standardized analgesia regimen, and preoperative education were strongly promoted by anesthesiologists and obtained the cooperation of surgeons.

Our patient's postoperative ambulation time decreased from an average of 6 postoperative hours in the first month of establishment to 4 h in 2024, while the time to drink was shortened from 6 h to 2 h, and the time to eat was shortened from 6 h to 4 h after surgery.

### 3.12 Discharge Assessment, Follow‐Up, and Quality Control

Our ASC uses the Postanesthesia Discharge Score (PADS) [44] to evaluate the discharge of patients. The PADS standard score is 10 points, and ≥ 9 points can be discharged from the hospital.

Our ASC conducts both manual and AI‐based phone follow‐ups for patients at 1, 3, 7, and 28 days postsurgery. The follow‐ups are carried out by nurses trained by anesthesiologists, and the results are entered into the DS management system, which can be accessed by anesthesiologists and surgeons at any time. To ensure medical safety, AI‐based phone follow‐ups are only conducted at 28 days postsurgery, as by then the patients' conditions have become very stable. The follow‐up content includes: wound pain, wound bleeding, changes in consciousness, nausea and vomiting, other clinical symptoms, whether to return for outpatient follow‐up, and timely guidance on complications. If serious complications occur, patients are advised to return to the hospital.

After monthly quality control meetings between anesthesiologists and surgeons, the average postoperative hospital stay was reduced from 10 to 6 h after surgery, and the readmission rate decreased from 2% to 0.22%.

## 4 Discussion

China has drawn on the experience of DS in Europe and the United States for nearly 100 years to walk out a road suitable for the development of DS in developing countries.

### 4.1 What is the Next Challenge Our Country Will Be Facing in Promoting DS and the Management Mode of Perioperative Period?

Most of the pioneers who performed DS, established DS wards, or ASCs in China did not have the conditions to go to Europe or the United States to learn how their doctors managed DS. The pioneers imitated and improved the management of DS in China by studying foreign literature. They were trying to gradually reduce what used to be a 2‐week hospital stay to 1 week, 5 days, 3 days, 48 h, 24 h, or even less.

Therefore, the evolution of the three management models of DS in China has a tendency to become centralized management. This is similar to the management models in developed countries. According to the data published in the United States, 80% of surgeries in the United States are currently performed in independent ASCs. Hospital‐owned ASCs in the United States account for only 3% of all ASCs.

The advantages of centralized management are efficiency, service improvement, and cost reduction, while the disadvantages are concerns about safety both in developed and developing countries. One of the key factors in ensuring the success of ASCs is the prudent selection of which patients are best suited to be cared for in an ASC, which are best cared for in DS in a regular hospital (with its extensive back resources), and which are most safely cared for as inpatients.

However, in China's current situation, it is still suitable for DS wards or centers to be attached to hospitals because developing countries are generally faced with the problems of a large population, low medical level, and insufficient government investment in medical care. Patients' distrust regarding primary health care structure leads to patients' concentration in large hospitals. Large hospitals in developing countries should expand the scale of DS to improve medical efficiency.

The management mode of perioperative period has gradually improved under the promotion of the exploration of medical pioneers and the support of government policies. Increasing the reimbursement ratio of national insurance for DS is also the next challenge our country will be facing in promoting DS.

China has a national unified government medical insurance with low premiums and basic medical benefits covering the entire population. Only a small number of people with better economic conditions will buy commercial insurance which is not provided by the government. China has a large population and, like developed countries, has entered an aging society. The government's insurance has limited funds. To ensure that patients with more serious conditions can receive treatment first, government insurance is used to only cover the expenses of inpatients. Currently, in developed regions of China, reimbursement for DS costs has been largely achieved, but in less‐developed areas, DS costs are still not reimbursed or the reimbursement ratio is lower than that for inpatients. The China Ambulatory Surgery Alliance has been working to make the government aware of the contribution of DS to reducing medical costs in order to persuade the government to increase the reimbursement ratio of national insurance for DS.

### 4.2 What Will the Role of Anesthesiologists be Going Forward?

We call on anesthesiologists, physicians, surgeons, nurses, and health system managers around the world to step up efforts to promote efficient, low‐cost DS in developing countries and thereby increase access to surgical treatment for the world's poor. DS requires anesthesiologists to participate in the whole process of patient management from preoperative preparation to postoperative recovery. The anesthesia management of DS is also a bridge and an important window for the transformation of anesthesiology to perioperative medicine.

Anesthesiologists can act as the directors and staff physicians of the DS ward or ASC, allowing the surgeons to concentrate only on performing the huge volume of surgeries in the operating room.

It is generally accepted that the important roles of anesthesiologists in preoperative anesthesia evaluation, operation, and anesthesia resuscitation are beyond doubt.

From the brief history of the development of DS in China, we can see that anesthesiologists quietly appeared in the ranks of the leaders of DS wards recently. However, we did not find any English literature that specifically reported that anesthesiologists were the directors and staff physicians of the DS ward or ASC.

If anesthesiologists can seize the opportunity, and strive to be directors and staff physicians in the DS ward or ASC, they will help to fully practice “preoperative anesthesia outpatient assessment–intraoperative precision anesthesia–postoperative anesthesia monitoring.” Only anesthesiologists who become leaders of the DS ward or ASC can promote their accurate management in terms of drive. For example, anesthesiologists will pay more attention to anesthesia‐related indicators that affect the outcome of patients undergoing DS, such as the rate of cancellation of anesthesia after entry, the rate of cancellation of surgery after anesthesia, the rate of intraoperative dental damage, the rate of severe reflux aspiration during anesthesia, the rate of severe allergy during anesthesia, the rate of known during general anesthesia, the rate of delayed transfer out of PACU, the rate of postoperative pain satisfaction, the incidence of hoarse voice after tracheal intubation and extubation under general anesthesia, the incidence of severe neurological complications after regional block anesthesia, etc.

Anesthesiologists also need to join the hospital‐level DS management committee or related organizations and participate in the unified management and quality control of DS throughout the hospital. Because for DS management, the operating room is a “black box.” Anesthesiologists who often work in the operating room can break through the blind area, and evaluate the problems of the surgical methods, the clinical level, and the communication ability of the operating doctors. And anesthesiologists are fair in this regard. Meanwhile, anesthesiologists are willing to work behind the scenes. So anesthesiologists have the characteristics of being the leaders of the DS ward or ASC.

### 4.3 What is the Next Task for the China Ambulatory Surgery Alliance?

In view of China's successful experience in promoting the development of DS, the IASS authorized the China Ambulatory Surgery Alliance to establish the “Asia DS Extension Training Center” for Asia and the “Africa DS Extension Training Center” for Africa in China in October 2016 and November 2018, respectively, to undertake the task of promoting DS in Asia and Africa.

## Author Contributions

J.L.: this author helped with conceptualization, literature search‚ and drafting the manuscript. C.X.: this author helped with conceiving and designing the project and drawing the images. D.F.: this author helped with resources and substantial manuscript revision.

## Ethics Statement

The authors have nothing to report.

## Consent

Manuscript is approved by all authors for publication.

## Conflicts of Interest

The authors declare no conflicts of interest.

## Data Availability

The authors have nothing to report.
